# Comparison of Normalization Methods for Analysis of TempO-Seq Targeted RNA Sequencing Data

**DOI:** 10.3389/fgene.2020.00594

**Published:** 2020-06-23

**Authors:** Pierre R. Bushel, Stephen S. Ferguson, Sreenivasa C. Ramaiahgari, Richard S. Paules, Scott S. Auerbach

**Affiliations:** ^1^Biostatistics and Computational Biology Branch, National Institute of Environmental Health Sciences of National Institutes of Health, Durham, NC, United States; ^2^Massive Genome Informatics Group, National Institute of Environmental Health Sciences of National Institutes of Health, Durham, NC, United States; ^3^Biomolecular Screening Branch, National Institute of Environmental Health Sciences of National Institutes of Health, Durham, NC, United States

**Keywords:** TempO-Seq, normalization, gene expression, mRNA, transcription

## Abstract

Analysis of bulk RNA sequencing (RNA-Seq) data is a valuable tool to understand transcription at the genome scale. Targeted sequencing of RNA has emerged as a practical means of assessing the majority of the transcriptomic space with less reliance on large resources for consumables and bioinformatics. TempO-Seq is a templated, multiplexed RNA-Seq platform that interrogates a panel of sentinel genes representative of genome-wide transcription. Nuances of the technology require proper preprocessing of the data. Various methods have been proposed and compared for normalizing bulk RNA-Seq data, but there has been little to no investigation of how the methods perform on TempO-Seq data. We simulated count data into two groups (treated vs. untreated) at seven-fold change (FC) levels (including no change) using control samples from human HepaRG cells run on TempO-Seq and normalized the data using seven normalization methods. Upper Quartile (UQ) performed the best with regard to maintaining FC levels as detected by a limma contrast between treated vs. untreated groups. For all FC levels, specificity of the UQ normalization was greater than 0.84 and sensitivity greater than 0.90 except for the no change and +1.5 levels. Furthermore, K-means clustering of the simulated genes normalized by UQ agreed the most with the FC assignments [adjusted Rand index (ARI) = 0.67]. Despite having an assumption of the majority of genes being unchanged, the DESeq2 scaling factors normalization method performed reasonably well as did simple normalization procedures counts per million (CPM) and total counts (TCs). These results suggest that for two class comparisons of TempO-Seq data, UQ, CPM, TC, or DESeq2 normalization should provide reasonably reliable results at absolute FC levels ≥2.0. These findings will help guide researchers to normalize TempO-Seq gene expression data for more reliable results.

## Introduction

Over the past 25 years, interrogation of genome-wide gene expression has taken many forms. cDNA and oligonucleotide microarrays (Millen and Glauser, 1978; Lockhart et al., 1996) analysis methods matured over time whereby preprocessing of the data for single-channel microarrays ultimately defaulted to the *de facto* Robust Multichip Average (RMA) normalization (Irizarry et al., 2003a, b). The advent of massive parallel signature sequencing (MPSS) and next-generation sequencing by synthesis for mRNA (RNA-Seq) ushered in a new paradigm for whole transcriptome analysis (Bainbridge et al., 2006; Cloonan et al., 2008; Lister et al., 2008; Mortazavi et al., 2008; Nagalakshmi et al., 2008). Crowdsourcing bioinformatics analysis of RNA-Seq data through the US Food and Drug Administration MicroArray Quality Control (MAQC), SEquence Quality Control (SEQC) phase effort led to a comprehensive assessment of RNA-Seq analysis including comparison to microarray and normalization using External RNA Control Consortium (ERCC) spike-in controls (Consortium, 2014; Risso et al., 2014; Wang et al., 2014; Xu et al., 2014). In addition, several studies have compared various normalization approaches for RNA-Seq data (Dillies et al., 2013; Zyprych-Walczak et al., 2015; Lin et al., 2016; Li et al., 2017). Proper normalization of gene expression data is essential to ensure valid and reliable results from downstream analyses (Park et al., 2003).

In the last few years, targeted sequencing of RNA has emerged as a practical means of capturing the totality of the transcriptomic space with less reliance on large resources for consumables and bioinformatics (Li et al., 2012). The TempO-Seq^TM^ technology from BioSpyder^TM^ is a templated, multiplexed RNA-Seq platform that measures the expression of sentinel genes representative of genome-wide transcription (Yeakley et al., 2017; Mav et al., 2018). A few advantages of TempO-Seq over RNA-Seq is that it does not require RNA purification, cDNA synthesis, nor capture of targeted RNA. In addition, by nature of the technology, it lacks 3′ end bias. Recently, several studies utilized the TempO-Seq platform for whole transcriptome profiling, primarily for toxicogenomics (Grimm et al., 2016; House et al., 2017; Yeakley et al., 2017; Bushel et al., 2018; Limonciel et al., 2018; Chappell et al., 2019; Ramaiahgari et al., 2019; Simon et al., 2019), but also carcinogenomics (Batai et al., 2018; Hanke et al., 2018), and to profile formalin-fixed paraffin-embedded (FFPE) tissue (Trejo et al., 2019). However, there has not been a comprehensive comparison of normalization methods applied to TempO-Seq data.

Here we utilize control samples from human HepaRG cells interrogated on the TempO-Seq platform to simulate gene expression data at seven-fold change (FC) levels including no change and normalized data using seven normalization methods for comparison. We show that based on sensitivity and specificity performance measures as well as the adjusted Rand index (ARI) as a measure of agreement, Upper Quartile (UQ) performed the best with respect to maintaining absolute FC levels ≥2.0 as detected in a two-group comparison. Counts Per Million (CPM), Total Counts (TCs), and DESeq2 normalization methods also performed reasonably well. The importance of this study is centered on providing the research community an assessment of which method to use for normalization of TempO-Seq data to ensure the reliable results from downstream analyses.

## Materials and Methods

### Cell Culture

HepaRG cells (Lonza, Catalog: NSHPRG) in cryopreserved form were thawed and seeded at approximately 20,000 cells/well onto collagen(I)-coated 384-well plates (Corning, Catalog #356667). Differentiated HepaRG cultures (2D-DIFF) were re-differentiated from cryopreserved suspension form over 10 days prior to vehicle exposures. Proliferated HepaRG cultures (PROLIF) were seeded at approximately 2,000 cells/well, grown for 3 days, and proliferated during vehicle exposures. For this, HepaRG were plated using William’s E medium (ThermoFisher, Catalog: A1217601) which was supplemented with MHPIT maintenance additive (Lonza, Catalog: MHPIT). Vehicle exposures durations were 96 h, and at the final time point, incubation media were removed and cultures were washed once with 50 μl phosphate-buffered saline (ThermoFisher, Catalog #10010023). Cells were subsequently lysed for high-throughput transcriptomics using 20 μl of 1 × Tempo-Seq lysis buffer (Biospyder) with a 15-min room temperature incubation with subsequent freezing at −80°C. Edge effects were minimized by excluding lysates in rows A, B, O, P, and Columns 1, 2, 23, and 24.

### TempO-Seq Analysis

Tempo-Seq analysis was performed as previously described by Biospyder, Inc. (Yeakley et al., 2017). Briefly, frozen lysates were thawed, and sequencing libraries for targeted panels of transcripts were generated. Each detector oligonucleotide (DO) consisted of complementary sequence to specific mRNA targets plus a universal primer binding site. Ligation of detector oligonucleotides *via* PCR amplification introduces adaptors required for sequencing and well-specific “barcodes” that link sequencing data to a specific well of origin. Barcode sequences flank the target sequence and are inserted into standard illumina adaptors to permit dual-index sequencing and deconvolution of sample-specific reads using standard illumina software. All PCR-amplified and barcoded samples were pooled into a single library for sequencing on a HighSeq 2500 sequencer (Illumina Inc., San Diego, CA) using a 50 cycle single-end read flow cell. Processing of the sequencing data was conducted using Illumina’s bcl2fastq software employing default parameter settings allowing for 1 mismatch per read. Sequencing reads were de-multiplexed using standard instrument software for each sample using barcodes to give FASTQ files linked to each well. Downsampled data was generated to obtain 500 mapped reads per gene on average. The 50 bp reads in the fastq files were aligned using bowtie version 1.2.2 (using parameters: -v 3 -k 1 -m 1 –best –strata –trim3 1) to a manifest of the TempO-Seq target genes sequences (a subset of the human transcriptome [Refseq release 70 downloaded July 23rd 2015)] reflecting the 50 bp sequences targeted by the DOs). The utility “idxstats” in the samtools package^1^ was used to generate read count data matrices.

### Simulated TempO-Seq Data

Let *Y*_gs_m_ denote the read count of a gene *g* ∈{1,…,*G*} belonging to a group *m*∈{1,2} of a sample *S*∈{S_1_, S_2_,…,S_*N*_} such that:

$$Y_{g\,s_{m}}∼\text{NB}\left(\text{mean}=\mu_{g\,s_{m}},\mathrm{var}=\mu_{g\,s_{m}}\left(1+\frac{\mu_{g\,s_{m}}}{\theta_{g\,s_{m}}}\right)\right)$$

where θ_gs_m_ is the parameter measuring the dispersion in the data, μ_gs_m_is the true mean of the data, and NB is the negative binomial distribution. We set the dispersion parameter for each gene to be the same for all samples. Thus, θ_gs_m_ = θ_g__. Here,

$$\mu_{g\,s_{m}}=E\,\left[Y_{g\,s_{m}}\right]=\frac{\lambda_{g\,s_{m}}}{\sum_{g=1}^{G}\lambda_{g\,s_{m}}}\,M_{s_{m}}$$

where *M*_*s_m*_ is the sequencing depth for the *m*th group in sample *S*. *M*_*s_m*_ = 1 × 10^6^ *U*_*s_m*_ for *U*_*s_m*_∼Unif[0.2,1.5] where Unif is the uniform distribution. The bounds (minimum and maximum limits) of Unif and the estimation of the sample mean $\lambda_{{\mathrm{gs}}_{m}}^{*}$ and θ_g_ were obtained from a DESeq analysis (Anders and Huber, 2010) of TempO-Seq count data (Supplementary Data 1: 2,680 genes in the 75th percentile of counts and 240 HepaRG control cells in the 90th percentile of counts) generated as previously described (Ramaiahgari et al., 2019) where the read depth was down-sampled to approximately 500 mapped read counts per transcript (Yeakley et al., 2017). Then, to generate sets of simulated genes across the groups of samples, we defined $\lambda_{{\mathrm{gs}}_{m}}=\gamma_{{\mathrm{gs}}_{m}}\,\lambda_{{\mathrm{gs}}_{m}}^{*}$, where *S*_1_ is considered the untreated group of samples, *S*_2_ is considered a group of samples with a particular perturbation (treated) and the ratio

$$\gamma_{g\,s_{m}}=w_{g\,s_{1}}\,/\,w_{g\,s_{1}}.$$

The differential expression was simulated at different levels for the respective sets of genes using values of *w*_gs_m_ denoted in Table 1. For each FC set, 50 genes were simulated and for each group *m*, 35 samples were generated (Supplementary Data 2).

**TABLE 1:** Parameter settings to simulate data FC.

***m***			
**Set**	**1**	**2**	**FC**
A	6	1.5	−4
B	3	1.5	−2
C	2.25	1.5	−1.5
D	1.5	2.25	+1.5
E	1.5	3	+2
F	1.5	6	+4
G	1.5	1.5	0

*Parameters for the simulation model: *N* = 50 genes per FC set, 35 samples per *m* group, library size lower limit = 0.2 × 10^6^, and library size upper limit = 1.5 × 10^6^. FC, fold change.*

### Normalizations

The following normalizations were applied to the simulated data. Since the TempO-Seq platform is designed of genes that capture greater than 90% of the biological pathways, most of the genes are likely to be differentially expressed. Thus, we normalized the data using the following methods:

#### Total Counts

The counts per gene were normalized to TCs by dividing it by the total number of mapped reads per sample and multiplying by the mean total count across all the samples (Dillies et al., 2013). The TC normalized data were then transformed with log_2_ using an offset of 1.

#### Counts per Million

The counts per gene were normalized to CPM by dividing it by the total number of mapped reads per sample and multiplying by 1 × 10^6^ (Robinson et al., 2010). The CPM normalized data were then transformed with log_2_ using an offset of 1.

#### Median

The counts per gene were Median normalized (Dillies et al., 2013) by dividing it by the median of mapped reads for all the samples and multiplying by 1 × 10^6^. The Median normalized data were then transformed with log_2_ using an offset of 1.

#### Quantile

The counts per gene were quantile (Q) normalized using the *normalizeQuantiles* function in the Bioconductor package limma (Ritchie et al., 2015). The method normalizes the counts of the genes in a sample to have the same quantiles across the samples in the data set. If there are ties among the genes for a particular sample, then the ties are normalized to the same value (i.e., the average of the quantiles for the tied values).

#### Upper Quartile

The counts per gene were UQ normalized using the *calcNormFactors* function in the Bioconductor package EdgeR (Robinson et al., 2010; Mccarthy et al., 2012) using the 75th percentile of the read counts that are mapped per sample. These scaling factors are then used to adjust the total mapped reads count for each sample.

The following normalization methods have an assumption that the majority of the genes on the platform are unchanged.

#### Trimmed Mean of M Values

The counts per gene were normalized using the “weighted” Trimmed Mean of M-values (TMM) approach (Robinson and Oshlack, 2010) in the Bioconductor package EdgeR. After trimming the data [5% for the A values, log ratio 0.3 for the M values to a reference array (the library whose upper quartile is closest to the mean upper quartile)], scaling factors for each sample were generated using the *calcNormFactors* function. Scaling factors were then used to adjust the total mapped reads count from each sample.

#### DESeq2

The counts per gene were normalized using the *estimateSizeFactors* function in the Bioconductor package DESeq2 (Love et al., 2014). The counts for each gene in each sample is divided by the geometric mean of the gene across all samples. The median of the ratios for the genes in a sample is the estimated size “scaling” size factor used to adjust the total mapped reads count from each sample.

### Performance of the Normalizations

#### Sensitivity

Sensitivity is the probability of the normalization of the read counts maintaining the genes’ limma-derived FC values.

Sensitivity=True⁢Positives/(True⁢Positives+False⁢Negatives).

#### Specificity

Specificity is the probability of the normalization of the read counts not falsely altering genes’ limma-derived FC values.

Specificity=True⁢Negatives/(False⁢Positives+True⁢Negatives).

#### Precision

Precision is the proportion of limma-derived FC values predicted correctly.

Precision=True⁢Positives/(True⁢Positives+False⁢Positives).

For multiclasses represented by the FC levels, sensitivity, specificity, and precision were calculated in one vs. all others fashion by comparing each FC level to the others combined.

#### Overall Accuracy

Accuracy is the proportion of limma-derived FC values predicted correctly over all the levels.

$$\text{Accuracy}=\frac{\left(\mathrm{True}\,\mathrm{Positives}+\mathrm{True}\,\mathrm{Negatives}\right)}{\left(\mathrm{True}\,\mathrm{Positives}+\mathrm{True}\,\mathrm{Negatives}+\mathrm{False}\,\mathrm{Positives}+\mathrm{False}\,\mathrm{Negatives}\right)}$$

#### Adjusted Rand Index

Validation of K-means (cosine dissimilarity metric and K = 7 FC sets) cluster assignment by any of the normalizations (log_2_ ratio of treated to average of the untreated with an offset of 1) was carried out using the ARI (Hubert and Arabie, 1985; Jain and Dubes, 1988; Yeung et al., 2001)

$$R^{\prime }=\frac{\sum_{i,j}\left(\begin{array}{c} n_{i\,j}\\ 2 \end{array}\right)-\left[\sum_{i}\left(\begin{array}{c} n_{i.}\\ 2 \end{array}\right)\,\sum_{j}\left(\begin{array}{c} n_{.j}\\ 2 \end{array}\right)\right]/\left(\begin{array}{c} n\\ 2 \end{array}\right)}{\frac{1}{2}\,\left[\sum_{i}\left(\begin{array}{c} n_{i.}\\ 2 \end{array}\right)+\sum_{j}\left(\begin{array}{c} n_{.j}\\ 2 \end{array}\right)\right]-\left[\sum_{i}\left(\begin{array}{c} n_{i.}\\ 2 \end{array}\right)\,\sum_{j}\left(\begin{array}{c} n_{.j}\\ 2 \end{array}\right)\right]/\left(\begin{array}{c} n\\ 2 \end{array}\right)}$$

where *n*_*ij*_ is the number of objects that are in both class *u*_*i*_ and cluster *v*_*j*_ of the *U* and *V* partitions, *n*_*i*_. and *n*.*_*j*_* are the number of objects in class *u*_*i*_ and cluster *v*_*j*_, respectively, and *n* is the total number of objects (*n*). *R*′ ranges between 0 and 1. *R*′ = 1 when the two partitions agree 100% and *R*‘ = 0 when the two partitions are selected by chance.

## Results

### Simulation of Fold Change Data

Using the RNA extracted from 240 wells containing HepaRG control samples and interrogated on the TempO-Seq platform, we simulated count data for treated (perturbed) and untreated groups (35 samples in each) using a negative binomial distribution with mean and dispersion estimates randomly sampled from the sentinel genes in the control samples. Table 1 lists the parameters used to generate seven FC sets of genes (A – G) from a comparison of group *m* = 2 vs. *m* = 1 denoting simulated treated samples vs. untreated samples. Sets A - F representing -4, -2, -1.5, +1.5, +2, and +4 FC, respectively, contain 50 simulated genes each, while set G with 50 simulated genes also represents no FC. The proportion of genes satisfies the assumption that the majority of genes are differentially expressed in a perturbed biological system with samples assayed on the TempO-Seq targeted platform. Thus, the comparisons of the normalization approaches would be more representative of the FC distribution of the genes in a typical targeted TempO-Seq analysis.

**FIGURE 1:**
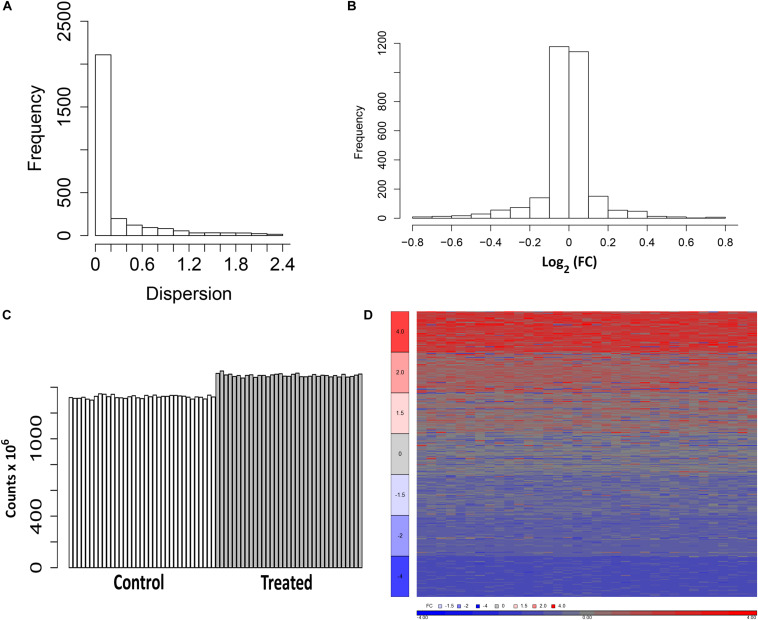
Representations of the estimates of the fold change (FC) values and simulation of data. **(A)** Dispersion from the DESeq negative binomial model. Dispersions less than or equal to 3.0 are plotted. **(B)** Distribution of the log_2_ FC estimated from a binomial test of the control samples randomly assigned to two groups (control1 and control2). Values within the range [-0.8, +0.8] are plotted. **(C)** Mapped reads of the samples from the simulation (untreated and treated). **(D)** Heat map of the log_2_ FC (ratio of treated to average of the untreated with an offset of 1) from 350 simulated genes (50 from each set in Table 1).

As shown in Figure 1A, the majority of the transcripts from the control samples have dispersion estimates less than 0.2 and thus have variances close to the mean. The 2,680 fitted genes from a binomial test of control samples randomly divided into two groups have predominantly a log_2_ FC approximately equal to 0 (Figure 1B). The heterogeneity of the 240 HepaRG samples is observed by the differential expression of some of the genes between the two groups of controls. Following the perturbations to generate the FC sets, the read depth of the samples ranged between 1.3 × 10^6^ and 1.35 × 10^6^ for the untreated and 1.47 × 10^6^ and 1.53 × 10^6^ for the treated (Figure 1C). The FC range and variability of the genes modeled in the simulation are visualized in a heat map diagram (Figure 1D).

### Negligible Normalization Impact on the Distribution and Variance of the Simulated Data

We used seven common normalization methods for RNA-Seq data to compare the effect on the simulated data. Five normalization methods, UQ, Median, CPM, TCs, and Q, adjust the data within sample, whereas two normalization methods, TMM and DESeq2, adjust the data within and between samples. The two latter methods have assumptions that the majority of the genes are not changed. This does not typically hold true for TempO-Seq data since the platform is designed with sentinel genes that capture the totality of the transcriptomic space (Mav et al., 2018). Despite a possible violation of the assumption, we included these two methods in the comparison since at least one has been recently used on TempO-Seq data (House et al., 2017).

**FIGURE 2:**
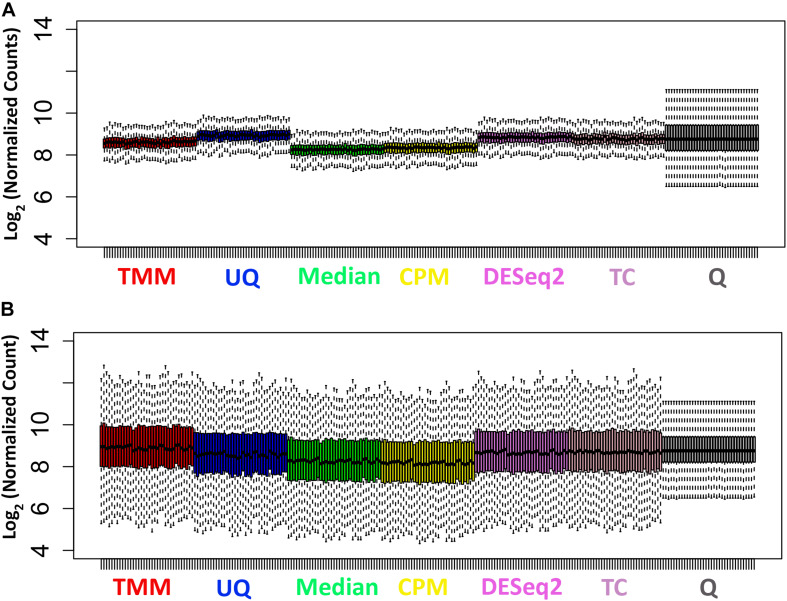
Distribution of normalized data. **(A)** Untreated samples. **(B)** Treated samples.

As shown in Figure 2A, the distributions of the log_2_ normalized count data for the untreated samples are relatively tight with short whiskers and consistent across the samples. The Q normalization appears to remove a fair amount of the variability across the samples. The Median and CPM normalized data shift the median of the data lower than the other normalizations. The distributions of the log_2_ normalized count data from the treated samples are more variable with longer whiskers (Figure 2B). The median values of the samples are more consistent across the normalizations. The Q normalized data have the same effect as it did with the untreated samples.

**FIGURE 3:**
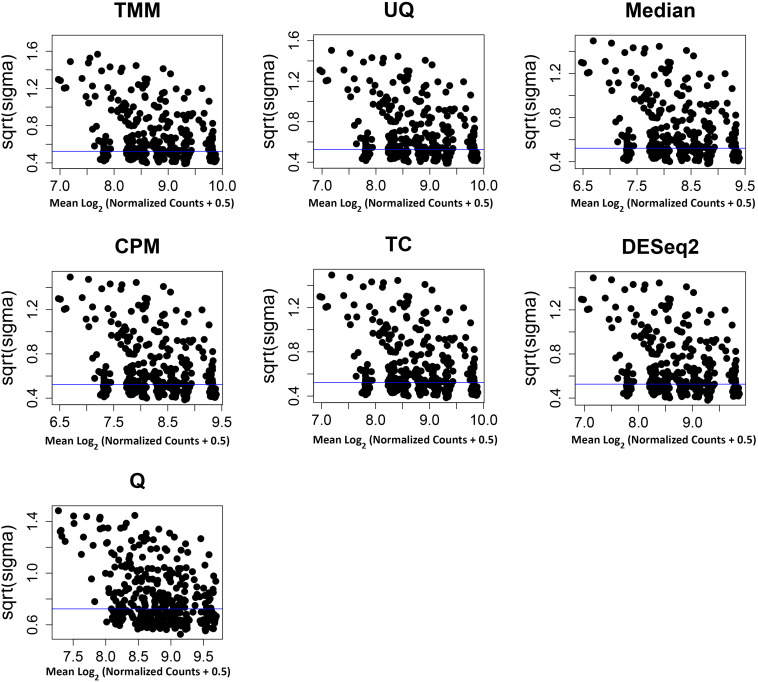
Mean–variance relationship of normalized data. The x-axis is the average of the log_2_ (normalized data + 0.5), and the y-axis is the square root of the variance from limma linear model of the normalized data. The y-axes have different scales. The average residual standard deviation is marked by a horizontal blue line.

**FIGURE 4:**
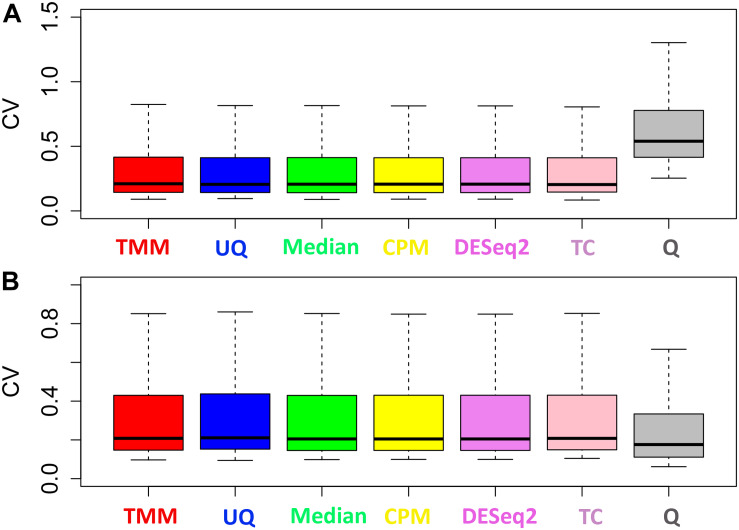
Distribution of the coefficient of variation. **(A)** Untreated samples. **(B)** Treated samples. The y-axes have different scales.

The normalization methods had no observable effect on the mean–variance relationship of the data (Figure 3). Only the Q normalization exhibited a lower average of the residual standard deviation (horizontal blue line) compared to the other normalization methods. In addition, the coefficient of variation (CV) of the data by normalization method also did not vary either except for Q (Figure 4). The CV for the Q normalization was higher that the other normalizations for the untreated samples but relatively the same for the treated samples.

### Normalization Impact Affecting Fold Change

**FIGURE 5:**
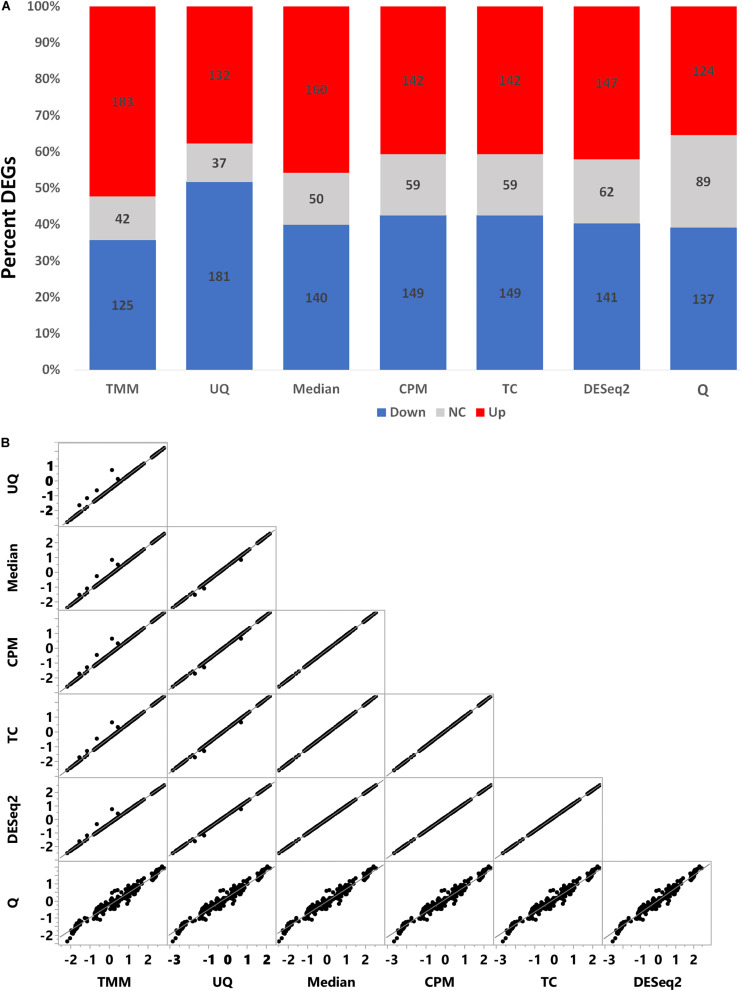
Comparison of limma fold change (FC) values. **(A)** Percent of differentially expressed genes as upregulated (red), downregulated (blue), or no change (gray) from the limma model comparing treated to untreated. **(B)** Correlation of log_2_ FC values from the limma model.

To assess the effect of normalization on the FC estimate of the simulated data, we used limma to test the comparison of treated vs. untreated. The percent of up and down differentially expressed genes varied according to normalization (Figure 5A). TMM normalization had more upregulated genes than downregulated genes, whereas the converse was true for UQ which had more downregulated genes than upregulated genes. CPM, TC, and DESeq2 had relatively the same proportion of differential genes. Q normalization had the most non-changed genes, while Median normalization had approximately the expected proportion of up, down, and non-changed genes.

The limma FC estimates for each normalization were then binned as follows to compare the similarity of the normalizations:

bin-4:(∞,-3.0],bin-2:(-3.0,-2.0],

bin:-1.5⁢(-2.0,-1.5],bin⁢0:(-1.5,+1.5),

bin+1.5:[+1.5,+2.0),bin+2:[+2.0,+3.0),

bin+4:[+3.0,∞).

As shown in Figure 5B, the log_2_ FC estimates from limma for each normalization method correlated very well with each other except with Q normalization. Although the Pearson correlation (*r*) values are greater than +0.98 (Table 2), there are visually some simulated genes with FC values that are impacted differently by the normalizations (Figure 5A). Q normalization has the greatest impact on the FC values.

**TABLE 2:** Correlations of log_2_ FC from limma.

	**TMM**	**UQ**	**Median**	**CPM**	**TC**	**DESeq2**	**Q**
TMM	1	0.997734	0.998682	0.998683	0.998683	0.998680	0.984968
UQ	0.997734	1	0.999782	0.999782	0.999782	0.999783	0.985386
Median	0.998682	0.999782	1	1.000000	1.000000	1.000000	0.985765
CPM	0.998683	0.999782	1.000000	1	1.000000	1.000000	0.985765
TC	0.998683	0.999782	1.000000	1.000000	1	1.000000	0.985765
DESeq2	0.998680	0.999783	1.000000	1.000000	1.000000	1	0.985764
Q	0.984968	0.985386	0.985765	0.985765	0.985765	0.985764	1

*CPM, counts per million; FC, fold change; Q, quantile; TC, total count; TMM, Trimmed Mean of M-values; UQ, upper quartile.*

In terms of the performance of the normalizations maintaining the expected FC level, UQ, CPM, TC, and DESeq2 normalizations had high sensitivity (greater than 0.840) at absolute FC levels greater than or equal to 2.0 (Table 3). Other normalizations performed better sensitivity-wise at either end of the FC spectrum. Specificity of the normalizations was reasonably high for all of the methods. DESeq2 had the highest accuracy overall (0.694) in maintaining the FC levels, followed by all the other methods. Q normalization which had the worst accuracy = 0.269. The precision of the normalization methods revealed that all performed well at either one or both extremes of the FC spectrum, but Median and UQ normalization methods were more precise at most FC levels.

**TABLE 3:** Performance of normalizations.

	**FC**	**CPM**	**TC**	**Median**	**Q**	**UQ**	**DESeq2**	**TMM**
Accuracy	*Overall*	0.688	0.688	0.683	0.269	0.683	0.694	0.566
Sensitivity	–4	0.980	0.980	1.000	1.000	0.962	0.980	1.000
	–2	0.950	0.950	0.600	0.070	0.898	0.947	0.105
	–1.5	0.776	0.776	0.115	0.114	0.915	0.326	0.000
	0.0	0.461	0.461	0.475	0.287	0.448	0.461	0.372
	1.5	0.174	0.174	0.840	0.154	0.045	0.653	0.833
	2.0	0.846	0.846	0.955	0.108	1.000	0.941	0.652
	4.0	1.000	1.000	1.000	1.000	1.000	1.000	0.907
Specificity	–4	1.000	1.000	0.997	0.888	1.000	1.000	0.949
	–2	0.961	0.961	0.864	0.847	0.980	0.903	0.855
	–1.5	0.960	0.960	0.852	0.854	0.977	0.885	0.846
	0.0	0.988	0.988	0.992	0.989	0.988	0.988	0.978
	1.5	0.862	0.862	0.973	0.859	0.843	0.940	0.922
	2.0	0.884	0.884	0.974	0.853	0.867	0.943	0.975
	4.0	0.997	0.997	0.997	0.901	0.997	0.997	0.997
Precision	–4	1.000	1.000	0.980	0.240	1.000	1.000	0.680
	–2	0.760	0.760	0.060	0.060	0.880	0.360	0.040
	–1.5	0.760	0.760	0.120	0.080	0.860	0.300	0.000
	0.0	0.940	0.940	0.960	0.960	0.940	0.940	0.900
	1.5	0.160	0.160	0.840	0.120	0.040	0.640	0.500
	2.0	0.220	0.220	0.840	0.080	0.080	0.640	0.860
	4.0	0.980	0.980	0.980	0.340	0.980	0.980	0.980

*CPM, counts per million; FC, fold change; Q, quantile; TC, total count; TMM, Trimmed Mean of M-values; UQ, upper quartile.*

To test the agreement of the normalized data with the FC group assignment, we K-means clustered the genes using the log_2_ ratio values of the treated to the average of the untreated with K = 7. The ARI measures the amount of agreement between the genes in the clusters and their FC set assignment. The ARI ranges from 0 to 1, where 0 defines the agreement is essentially random, and 1 indicates that the agreement is perfect. As shown in Table 4, UQ normalization had the highest ARI score of 0.67, followed by TC, CPM, DESeq2, and then Median. TMM normalization was subpar in agreement, and not surprisingly, Q normalization had the worst agreement overall.

**TABLE 4:** Agreement with FC assignment.

**Normalization**	**ARI**
TMM	0.55
UQ	0.67
Median	0.60
CPM	0.63
TC	0.65
DESeq2	0.62
Q	0.30

*Groups of genes created by K-means (K = 7) clustering of simulated genes using log_2_ of normalized ratio values (treated to average of untreated) with an offset of 1 and the cosine dissimilarity metric. ARI, adjusted Rand index; CPM, counts per million; FC, fold change; Q, quantile; TC, total count; TMM, Trimmed Mean of M-values; UQ, upper quartile.*

## Discussion

The various gene expression platforms that researchers rely on for whole genome transcriptomics have their own *de facto* normalization method that are preferred by analysts. With single-channel microarray, RMA became the standard normalization approach. For bulk RNA-seq, many users gravitated to DESeq2 for normalization. For single-cell RNA-Seq, the path forward continues to emerge. In the case of newer platforms such as TempO-Seq targeted RNA sequencing, there has not been an evaluation of the performance of several normalization methods. Recent publications using the TempO-Seq platform used DESeq2 or CPM for normalization (Grimm et al., 2016; House et al., 2017; Yeakley et al., 2017; Batai et al., 2018; Bushel et al., 2018; Hanke et al., 2018; Limonciel et al., 2018; Chappell et al., 2019; Ramaiahgari et al., 2019; Simon et al., 2019). In this study, we compared seven normalization methods using simulated data from human HepaRG control cells to determine which methods maintained genes at seven assigned FC levels (Table 1). We found that based on sensitivity, specificity, precision and accuracy performance metrics (Table 3) as well as the ARI that assessed the FC group assignments (Table 4), UQ at the 75th percentile of genes performed best. UQ performed well in comparisons of bulk RNA-Seq normalization when it is scaled across the samples (Li et al., 2017). This added adjustment might be necessary for bulk RNA-Seq and not TempO-Seq as the former would likely have more varying read depths since the majority of genes are not changed. TempO-Seq, by nature of the targeted platform content, is designed with sentinel genes that capture the predominance of the transcriptional landscape. Despite the assumption that the majority of the genes are not changed, DESeq2 performed reasonably well in our TempO-Seq normalization comparison as did more simple normalization methods such as CPM and TCs.

Our analysis reveals some interesting findings regarding some of the statistics surrounding TempO-Seq normalized data. For instance, the dispersion of the genes used to generate random variates for normalization is typically less than 0.2 (Figure 1A). This suggests that at least for the control HepaRG cultures, the variance of the genes is close to the mean. Therefore, variance stabilization transformations might not be necessary to preprocess the data. Another interesting finding is that most of the genes in the control HepaRG wells do not vary much between replicates. The majority of the genes from the simulated data have a log_2_ FC approximately equal to 0 (Figure 1B). If the expression of the genes between the control samples varied more, it would be more challenging to control the Type I error. We also found that the normalization methods, except for Q, did not affect the distribution, variance, or coefficient of variation (CV) of the data (Figures 2–4). Previous studies on the comparison of normalization methods for bulk RNA-Seq agree with our finding regarding the distribution of the data and some of the summary statistics (Dillies et al., 2013; Lin et al., 2016).

Our investigation does have some limitations that are important to keep in mind. UQ, CPM, TC, and DESeq2 performed reasonably well in normalizing the simulated TempO-Seq data at an absolute FC level ≥2.0, with UQ being the best overall. Below that threshold, the performances suffered. Furthermore, our analysis is for a two-class comparison of groups. More investigation is needed to determine which normalization is superior when comparing more groups or data with a dose or time dependency. Our analysis focused on simulated data from control HepaRG wells and not treated cells. We simulated various FC levels to mimic perturbations, but we assumed that the dispersion for each gene was the same across the two groups. It may be the case that genes in a perturbed system might have dispersion and other statistical properties different from the control cells which may presumably impact the normalization. Note that the TempO-Seq platform that we elevated the normalization methods is a targeted, human version with about 3,000 probes/transcripts as content. Normalizations for data from whole genome TempO-Seq platforms and/or targeted content from other species may perform differently. Finally, the simulated data used DESeq, the previous version of DESeq2, for estimating the dispersion and mean of the genes. This may have provided an unfair advantage to the DESeq2 performance in the comparison. Despite the aforementioned limitations, our results shed some light on the utilization of various standard methods for normalization of TempO-Seq data and that if used in a proper way, several choices will hopefully provide reliable analysis results. Future work will investigate more sophisticated normalization methods for TempO-Seq data and concentrate on data generated from whole genome platforms, other species’ gene content, and factorial or series experimental designs.

## Data Availability Statement

The original contributions presented in the study are included in the article/Supplementary Material, further inquiries can be directed to the corresponding author/s.

## Author Contributions

SF and SR generated HepaRG cultures and TempO-Seq data. PB designed the simulation study, implemented the computations, performed the analyses, and wrote parts of the manuscript. SF and SA provided interpretation of the results and wrote part of the manuscript. RP provided interpretation of the results. All authors contributed to the article and approved the submitted version.

## Conflict of Interest

The authors declare that the research was conducted in the absence of any commercial or financial relationships that could be construed as a potential conflict of interest.

## Footnotes

1 http://www.htslib.org
