# Erratum: Comparison of Normalization Methods for Analysis of TempO-Seq Targeted RNA Sequencing Data

**DOI:** 10.3389/fgene.2020.00943

**Published:** 2020-08-11

**Authors:** Frontiers Production Office

**Affiliations:** Frontiers Media SA, Lausanne, Switzerland

**Keywords:** TempO-Seq, normalization, gene expression, mRNA, transcription

Due to a production error, the equation for “Overall Accuracy” was typeset erroneously. The correct equation follows below:

$$\text{Accuracy}=\;\frac{\left(\text{True Positives }+\text{ True Negatives}\right)}{\left(\text{True Positives }+\text{ True Negatives }+\text{ False Positives }+\text{ False Negatives}\right)}$$

The publisher apologizes for this mistake. The original article has been updated.

